# The hunger-obesity paradox: Exploring food banking system characteristics and obesity inequities among food-insecure pantry clients

**DOI:** 10.1371/journal.pone.0239778

**Published:** 2020-10-21

**Authors:** Kristen Cooksey Stowers, Nana Yaa A. Marfo, Eminet Abebe Gurganus, Kim M. Gans, Shiriki K. Kumanyika, Marlene B. Schwartz

**Affiliations:** 1 Department of Allied Health Sciences, Storrs, Connecticut, United States of America; 2 Rudd Center for Food Policy and Obesity, University of Connecticut, Hartford, Connecticut, United States of America; 3 Department of Psychological Sciences, University of Connecticut, Storrs, Connecticut, United States of America; 4 Department of Human Development and Family Sciences, University of Connecticut, Storrs, Connecticut, United States of America; 5 Department of Community Health and Prevention, Drexel University Dornsife School of Public Health, Philadelphia, Pennsylvania, United States of America; Newcastle University, UNITED KINGDOM

## Abstract

**Purpose:**

Heightened obesity risk among food-insecure food pantry clients is a health equity issue because the co-occurrence of obesity and hunger is deeply-rooted in systematic social disadvantage and historical oppression. This qualitative study examined key stakeholders’ perspectives of the relationship between the U.S. food banking system and obesity disparities among food insecure clients.

**Methods:**

We conducted in-depth, semi-structured interviews with 10 key stakeholders (e.g., food bank director, food bank board member, advocate) who are familiar with food bank operations. Data were transcribed verbatim, coded in NVivo [v11], and analyzed using thematic analysis.

**Results:**

Multiple themes emerged drawing linkages between *structural characteristics* of the food banking system and disparities in the dual burden of food insecurity and obesity: [a] access to unhealthy food from donors; [b] federal emergency food policy and programming; [c] state-level emergency food policy and programming; [d] geography-based risk profiles; and [e] inadequate food supply versus client need. Interviewees also identified *social* challenges between system leaders and clients that maintain disparities in obesity risk among individuals with very low food security including: [a] media representation and stereotypes about food pantry clients; [b] mistrust in communities of color; [c] lack of inclusion/representation among food bank system leaders; and [d] access to information.

**Conclusion:**

Future efforts to alleviate obesity inequities among clients chronically burdened by food insecurity, especially among certain subpopulations of clients, should prioritize policy, systems, and environmental strategies to overcome these *structural* and *social* challenges within the food banking system.

## Introduction

According to the United States Department of Agriculture, 11.1% (14.3 million) of American households experience food insecurity with 4.3% (5.6 million) experiencing very low food security characterized by multiple indications of reduced food intake and disrupted eating patterns due to a lack of resources [1, 2]. Most households with very low food security also report experiencing hunger at some time during the year and skipping meals because there was not enough money for food [3]. Recent data from a national survey of US adults indicates that these disparities have significantly widened due to COVID-19, with 44% of low-income adults currently reporting food insecurity. Alarmingly, only 1 in 5 adults with very low food security was able to follow emergency guidelines to stock two weeks of food at a time [4]. Along with rising rates of food insecurity, there have been dramatic increases in obesity in the United States [U.S.] since the 1990’s [4]. The 2019 State of Obesity report revealed that over 40% of adults have obesity [5].

Inequitable obesity risk among women, racial-ethnic minorities, individuals with low income, and other historically marginalized populations is well-documented [6–9]. Further, people burdened by food insecurity—especially women and older adults—are at an increased risk of overweight and obesity, [10–24]. This phenomenon, in which obesity and food insecurity coexist within the same person or household, is frequently referred to as the hunger-obesity paradox or the food insecurity paradox [15–17]. The relationship between hunger and obesity is considered paradoxical because food insecurity is traditionally associated with underconsumption while obesity is commonly linked to overconsumption [15, 25, 26].

Past work aiming to understand the hunger-obesity paradox has largely focused on the role of federal food and nutrition assistance programs, including the Supplemental Nutrition Assistance Program [SNAP] [16, 24, 27–29]. For instance, a literature review on the role of SNAP concluded that increased budget allotments and altered budgeting cycles are warranted to ensure that households can afford not just any food but nutrient-dense foods throughout the entire month [16]. Franklin and colleagues [2012] conducted a literature review of factors that mediate the relationship between food insecurity and obesity. In addition to confirming a strong and positive relationship between food insecurity and obesity in women, their review revealed other important mediators, including SNAP participation, marital status, maternal stressors, and adolescence [29]. However, utilization of the food banking system (commonly referred to as the emergency food system or hunger relief network) has been understudied as a possible mediator.

The food banking system in the United States includes a nationwide network of 200 food banks that distribute food via community-based agencies including food pantries and meal programs [30–33]. In 2014, 46.5 million people (15% of the U.S. population) in 15.5 million households relied on foods distributed through the food banking system [34]. This population, frequently referred to as “clients,” is very diverse in terms of race, ethnicity, age, employment status, educational attainment, household size, and health conditions [e.g., diabetes, high blood pressure] [34–39]. Compared to the general U.S. population, the client population reflects higher proportions of individuals who are Black or multi-racial; lower rates of White or Asian people, and a similar proportion of individuals who are Hispanic [34]. Compared to the U.S. population, households that utilize the food banking system are also more likely to have children under age 18; have lower median incomes; and receive SNAP benefits [34].

Historically, the food banking system was designed to respond to sudden hunger crises on a temporary basis and not to disease prevention or treatment [31]. Increasingly, the system is evolving to respond to the additional concerns of obesity and other diet-related illnesses (e.g., increased produce distribution) [30, 31, 33]. Previous studies focusing on key stakeholders’ views of the food banking system document the aspects of food banking culture, capacity, policies, and practices that influence the nutritional quality of the foods distributed to clients [30, 33]. However, these studies do not address the issue of health inequities.

Inequities in obesity prevalence are observed not only when comparing food pantry clients to the broader U.S. population [40], but also when comparing people *within* the client population [39–42]. For example, Robaina and Martin’s study of pantry clients in Hartford revealed that female clients were more than 4 times as likely as male clients to have obesity. Further, their results showed that of those with obesity or severe obesity in the sample [n = 82], 78% were women [n = 64] [39]. Previous research also points to racial-ethnic health disparities within samples of pantry clients including a heightened risk for obesity among Latinx and Black participants (57.1% of Latinx vs. 40.2% of Black in vs. 37.8% of White participants) [42]. Kaiser and Cafer’s recent study of severe obesity among food pantry clients revealed that long-term pantry users with very low food security had significantly greater odds of having obesity than short term users with higher food security (OR = 1.68, p < .05) [40]. Previous studies also point to other sociodemographic differences in obesity rates, including a higher risk of obesity among clients who pay deductibles for private insurance, and a lower risk of obesity among clients who are older, unemployed, and enrolled in SNAP [39–42].

Heightened obesity risk among food pantry clients in general, and within subpopulations of clients in particular, is a health equity issue because the co-occurrence of obesity and hunger is deeply-rooted in systematic social disadvantage and historical oppression [8, 43–52]. Clients’ chronic dependence on food pantries and other community-based agencies as regular food sources [34, 36, 37, 39, 40, 46, 47, 52] raises the question of the potential influence of the food banking system on clients’ ability to control their weight or manage obesity-related conditions such as diabetes and hypertension. For many clients, food pantries provide about 25% of their groceries for an average of 3 years [47].

The purpose of this study was to examine key stakeholders’ views about the food banking system’s role in the co-occurrence of hunger and obesity inequities among clients. An equity-oriented systems approach [8, 45, 48–51] is warranted to identify the root causes of heightened obesity risk among subpopulations with food insecurity and to identify entry points for tailoring policies and programming.

## Methods

In-depth, semi-structured interviews were conducted between May and July of 2017. A purposive sample of ten key stakeholders was recruited through a referral process. Individuals were invited to participate if they were familiar with the food banking system in their professional capacity and matched a list of priority stakeholders and perspectives for the present study (e.g., food bank director, elected official, advocate, funder, researcher). At the end of each interview, key stakeholders were asked to suggest other professionals to invite for participation in the study. Two members of the research team (KCS and MBS) met to screen referrals based on the previously mentioned list of priority stakeholders. Key stakeholders were recruited until saturation was achieved (i.e., no additional themes were introduced) indicating high data quality and thoroughness in our exploration of the topic. All stakeholders invited to be interviewed agreed to participate. The sample included: a food bank board member; two food bank executive directors; a national anti-hunger organization leader; two foundation program officers; a community-engaged researcher; two anti-hunger advocates; and a State House representative.

The interviews were administered by one interviewer (KCS) who has extensive training and experience with conducting in-depth interviews and probing. Key stakeholders provided verbal consent before starting the interview and each session was audio recorded. The semi-structured interview protocol included a topic guide exploring the relationship between structural and social characteristics of the food banking system and health inequities among individuals experiencing food insecurity. Several questions about structural entry points were adapted from the Center for Social Inclusion’s guidance on “Asking Structural Questions to Identify Structural Entry Points” [48]. The interviewer probed for additional details about obesity disparities. The interviews were conducted via telephone and lasted from 45 to 120 minutes. The University of Connecticut Institutional Review Board reviewed and approved the research design and interview protocol. See Table 1 for the interview protocol.

**Table 1:** Factors contributing to hunger and obesity disparities among systems clients: Interview questions.

Factors Impacting Disparities	Relevant Interview Questions
**Structural Factors**	Name at least two (no more than five) structural inequities in the emergency/charitable food system that potential contribute to avoidable health disparities? (*e*.*g*., *Lack of access to healthy foods*, *more funding for farmers of color*, *fair wages for food system workers*.)
	Name at least three (and no more than six) institutions impacting the problem we are trying to solve. Note: These institutions may be impacting the problem by doing something wrong, doing something right, doing nothing or some combination of all three. (*e*.*g*., *Large companies like Monsanto*, *public schools*, *USDA*, *restaurant industry*)
	Name at least three policies that are impacting the problem(s) (if not obvious or redundant from the institutions named). (*e*.*g*., *Farm bill*, *immigration*, *minimum wage)*
	Which of the institutions and policies impact the ***root causes*** of the problem (*e*.*g*., *Low wages do not allow for low---income individuals to afford healthy foods; farm bill policies are not sufficient to invest in infrastructure needed to provide healthy foods to communities lacking access*.*)*
**Social Factors**	A 2011 report entitled “Advancing Equity within the Emergency Food Provider Network in Maricopa County” identified the following structural issues as barriers to accessing emergency/charitable foods for the Latino community in Arizona… Discrimination and racial bias Identification requirements Perception of Food Banks and Pantries as Government affiliated programs Differences in Language Access (Physical location and limited hours of operation)
	What are your thoughts on how they contribute to health disparities for Latinos, African Americans and other communities of color?
	What are some recommendations for addressing the barriers previously listed?

Audio recordings were transcribed verbatim, reviewed for errors, and analyzed using thematic analysis. Authors KCS and NYM read and reread the transcripts for familiarization and developed an initial coding guide through an iterative process. Two trained members of the research team (NYM and EAG) plus a third coder (KCS) applied the codes to the data using NVivo Statistical and Qualitative Data Analysis Software (v11).

After the first round of coding was completed, minor revisions were made to the coding guide. Coders (authors KCS, NYM, and EAG) reached adequate interrater reliability for the first three interviews (Cohen’s Kappa = .81) before coding the remaining interviews. The coding team met regularly to reconcile differences in how codes were being applied. Using a thematic analysis approach, three trained members of the research team (KCS, NYM, and EAG) extracted key themes and subthemes from the coded data. This process was led by a research team member (KCS) with expertise and methodological training in qualitative data analysis. Throughout the analyses, team meetings, memos, and notes were used to identify patterns of meaning and enhance conceptual interpretation. Major themes and subthemes were developed and refined using an iterative process of feedback sessions with the research team. To demonstrate the data-driven nature of the analytic findings, direct quotes from key stakeholders were extracted and are described in the following section.

## Results

As a whole, key stakeholders identified several structural and social inequities in the food banking system that perpetuate disparate risks of hunger and obesity among clients. Notably, the research team made a distinction between Structural and Social characteristics. Structural characteristics are ‘upstream’ factors that are institutionalized or organizational, whereas Social characteristics include attributes of the system that are interpersonal by nature (e.g., interactions between leaders and clients). As reflected in Tables 2 and 3, the following themes emerged.

**Table 2:** Structural characteristics perpetuating hunger and obesity among systems clients: Illustrative quotes.

Structural Theme	Representative Statement
**Access to unhealthy food from donors**	“I think the way the food bank system was designed, it was designed to take any surplus food that was available and distribute it… this long-held fear of losing donors—and by donors I mean corporate food and beverage donors… who are donating these large quantities, donations of food. You know, the good, the bad and the ugly.”
**Federal food assistance programming and policy**	“If food pantries receive food from the TEFAP program—temporary emergency federal assistance program—then they are required to have all of their clients sign a paper with their income eligibility… some food pantries will decide not to take TEFAP funding so that they don’t have to have all that paperwork for their clientele.”
**State-level food assistance programming and policy**	“We have a program called the state food purchase program—some states have these, some don’t. It’s basically like TEFAP but state dollars. There’s another program or policy rather that has been picking up momentum in some states around connecting dollars…providing dollars so that food banks can buy from local farmers.”
**Variation in risk across geography**	“As I talk with food banks around the country, some of them will feel like if they are not within an agricultural belt, geographically, that urban center where there’s not a lot of fresh produce being grown locally, they could be marginalized because there’s just fewer farmers locally who would think to donate to their food bank.”
**Food supply does not adequately meet nuanced client needs**	“There’s a lack there [in the food bank system] of getting certain very low sodium, very low fat, high fiber—the kind of things that the folks who are dealing with diabetes and heart disease and other health issues. When their doctor says, take with food, change your diet, maybe you have to be gluten-free now because we’re finding celiac disease, how can food banks respond and get the food that’s needed to not only serve a vulnerable population just in the way of food insecurity, but probably also a population that has some fairly specific food needs.”

**Table 3:** Social characteristics perpetuating hunger and obesity among systems clients: Illustrative quotes.

Social Theme	Representative Statement
**Media representation and stereotypes**	“There just seems to be a disconnect with people that aren’t hungry toward feeling like those that are hungry, it’s due to some uh…laziness on their part or, you know, it’s their problem not my problem.”
**Mistrust in communities of color**	“There’s a fear that as an illegal immigrant that you’re making yourself more vulnerable to being detained if you go over to a food bank or a food pantry. And probably, that perception I’m sure is higher among Latino immigrants because they’re the ones being flashed all across the media.”
**Inclusion, power, and representation**	“What are their [food pantries’] hiring practices if their staff is mostly white in a, you know, predominately Latino community? That may also be an issue.”
**Privilege of access to information**	“Those who are educated, they may not necessarily be wealthy, but those who are educated about the need to eat more healthfully—and they have the know-how on how to cook those items, those more wholesome items, too.”

### Structural inequities

#### Access to unhealthy food from donors

The data revealed a strong consensus among key stakeholders regarding the role of unhealthy food donations in contributing to health disparities. They recognized this as an artifact of the process through which the food banking system was established. Alluding to the distribution of surplus food as the primary function for which the system was intended, an anti-hunger advocate stated:

I think the way the food bank system was designed, it was designed to take any surplus food that was available and distribute it. And when that landscape changed, the environmental factors that we were faced with, the alarming rates of obesity and diabetes, and particularly the populations that are served through the food bank system are the more vulnerable to obesity and diabetes. …But you know, this long-held fear of losing donors—and by donors, I mean corporate food and beverage donors …who are donating these large quantities, donations of food. You know, the good, the bad and the ugly.

As illustrated by the quote above, key stakeholders related the prevalence of unhealthy food donations to food banks’ fear of losing corporate donors, hinting at the imbalance of power between the organizations that donate food and those that receive it.

#### Federal food and nutrition assistance programs

Multiple key stakeholders discussed the system’s reliance on support from the Temporary Emergency Federal Assistance Program (TEFAP). A community-engaged researcher pointed to administrative burden and required documentation as contributing to unequal access to TEFAP resources at the pantry level.

If food pantries receive food from the TEFAP program—temporary emergency federal assistance program—then they are required to have all of their clients sign a paper with their income eligibility, I think show ID, and show how many people are in their household. Food pantries don’t necessarily have to take food from TEFAP… some food pantries will decide not to take TEFAP funding so that they don’t have to have all that paperwork for their clientele.

An anti-hunger advocate noted how misconceptions about ID requirements for different food programs may deter eligible individuals from accessing foods distributed through the food banking system: “…yes they [clients] need their identification for the TEFAP program, for example. But it doesn’t have to be government-issued photo identification. You don’t need your social security number. [There are] misconceptions of what’s needed at that distribution point. So, I feel like there’s a lot of education that’s needed.”

A community-engaged researcher drew linkages between the implementation of ID-requirements and the current political climate’s “chilling effect” for undocumented immigrants needing to access emergency food.

So, you could imagine, for people who may be illegal immigrants… they have to show up to a church food pantry, and the church food pantry is asking them to fill out a government form asking how many people are in their household, asking about their income. With the current political climate, that could be quite frightening, and it may turn people away from trying to receive that food.

Key stakeholders acknowledged the SNAP program as having a major impact on food banking system clients, many of whom are program beneficiaries. A staff member of a national anti-hunger organization pointed out the health consequences of SNAP and food pantry resources running out before the end of the month:

If SNAP, if our emergency benefits as the federal government were as intended, meaning they pay enough money to help families get through the month, that would also, you know, lift some health disparities. As of right now, it’s just not happening for families; it’s still not enough. And they’re finding that they [clients] have to stretch their dollars, even with SNAP and food pantry assistance, so I think that leads to health disparities.

A food bank director pointed out the potential of leveraging SNAP initiatives like SNAP-Ed and fruits and vegetable incentive programs to address the co-occurrence of hunger and obesity among clients:

There are new programs called SNAP incentives that we think are good policies that help make healthy food more affordable. And then, I think there are lots of education programs out there too, so, in order for folks to be able to utilize the food they’re getting from the food bank, they can cook at home and learn about, you know, making healthier choices. Programs like the federal SNAP-Ed program we think is a very important component to all this.

#### State-level emergency food assistance programming and policy

A subset of key stakeholders discussed important state-level differences in additional resources to support food banks and policies to promote the increased distribution of fresh produce and other nutrient-dense food items. One food bank director shared:

In [state], we have a program called the state food purchase program—some states have these, some don’t. It’s basically like TEFAP but state dollars. There’s another program or policy rather that has been picking up momentum in some states around connecting dollars…providing dollars so that food banks can buy from local farmers. That has a health impact because that’s a lot of fresh fruits and vegetables and high-quality foods, so we like that policy.

#### Food banking system may contribute to geography-based risk profiles

In a few cases, key stakeholders acknowledged that place-based disparities in obesity risk may occur where clients in urban spaces chronically rely on food banks with limited access to farmers and fresh produce. A community-engaged researcher described how staff members of urban food banks across the country have noted this discrepancy:

You know, as I talk with food banks around the country, some of them will feel like if they are not within an agricultural belt, geographically, that urban center where there’s not a lot of fresh produce being grown locally, they could be marginalized because there are just fewer farmers locally who would think to donate to their food bank. So, for example when we were in [city], and we talked with [local] food banks, they just said they don’t have a lot of produce near the food banks. So, you know, perhaps in other geographic areas around the country where you have more farmers, who could naturally link up to the food banks, that might play a part.

#### Food supply does not adequately meet nuanced client needs

One recurrent theme that emerged in the interviews was the gap between the nutritional profile of the foods available in the food banking system and clients’ nutritional needs, particularly those with obesity, diabetes, and other diet-related health challenges. One foundation program officer commented:

There’s a lack of very low sodium, very low fat, high fiber [foods]–the kind of things that the folks who are dealing with diabetes and heart disease and other health issues [need]. When their doctor says, take with food, change your diet, maybe you have to be gluten-free now because we’re finding celiac disease, how can food banks respond and get the food that’s needed to not only serve a vulnerable population just in the way of food insecurity but probably also a population that has some fairly specific food needs.

Voicing the shared view across key stakeholders that clients need “culturally appropriate healthy food, not just any food”, a national anti-hunger organization staff member stated:

Just providing food to somebody who has diabetes isn’t going to give them a better health status just because they have food; it needs to be the appropriate, culturally sound food. Nutritious and recognizable, appropriate for their diet, that can really improve the health of the community.

Referring to clients that come into the food banking system with health conditions, key stakeholders also highlighted how the current system may contribute to worsening health outcomes. One foundation program officer noted, “…if the bank or the pantry doesn’t have those specific foods that they [clients] need to solve their health issues, then they’re just as bad—if not worse off. It’s almost probably making the health issue worse.”

### Social inequities

#### Media representation and stereotypes

When asked about potential social inequities within the food banking system, several key stakeholders noted how negative stereotypes about hunger and food insecurity permeate media representations and the broader sociopolitical conscience of the United States. In describing the indirect impact of these narrow views of clients of the food banking system, some key stakeholders identified an underlying disconnect between those who do and do not experience hunger and food insecurity. A foundation program officer stated, “…and there just seems to be a disconnect with people that aren’t hungry and those that are experiencing hunger, it’s due to some uh…laziness on their part or, you know, it’s their problem, not my problem.”

Multiple key stakeholders further highlighted how this disconnect and the resulting health disparities are perpetuated by the absence of face-to-face interaction between donors and recipients. A community-engaged researcher stated:

The donor can have these perceptions of… ‘oh they don’t want these things [healthy foods].’ How do you know that? You know? I think definitely, these structural entry points, that there aren’t as many opportunities for the donors and recipients to interact and have conversations. Then they would know actually there is a demand for healthier food.

#### Mistrust of the food banking system in communities of color

Communities of color’s mistrust and fear of being mistreated within the food banking system arose as a salient theme across interviews. Multiple key stakeholders highlighted how fear and mistrust of the food banking system may disproportionately impact immigrant communities in the U.S. Noting the role of the sociopolitical climate in driving this fear and mistrust among undocumented immigrants experiencing food insecurity, one national program officer said, “There’s a fear that as an illegal immigrant that you’re making yourself more vulnerable to being detained if you go over to a food bank or a food pantry. And probably, that perception I’m sure is higher among Latino immigrants because they’re the ones being flashed all across the media.”

A food bank director pointed out how the fear of deportation is not the only source of mistrust that might be deterring immigrants from engaging with the food banking system. Fear of racism and discrimination can also act as significant barriers. A national program officer shared: “But I would imagine it’s similar for any immigrant from any country that’s here, and probably even regardless of their legal status when you’re talking about racial discrimination and assumptions and that sort of thing.”

#### Inclusion, power, and representation

Some key stakeholders noted the implicit yet powerful role that whiteness may play in perpetuating health inequities in the food banking system. In situations where the staff and volunteers providing direct food assistance are predominantly White and recipients come from more racially and ethnically diverse backgrounds, an imbalanced power dynamic can take hold between food providers and recipients. Numerous key stakeholders pointed out the need for organizations within the system to reflect upon their hiring and recruiting practices.

In regards to food banking staff, a national program officer asked, “what are their hiring practices if their staff is mostly white in a, you know, predominately Latino community? That may also be an issue.” This program officer asked a similar question regarding volunteer recruitment: “What other businesses do they need to speak with about enlisting volunteers, or what other community organizations or what high schools can they go to try and get more volunteers of color in?”

More broadly, key stakeholders suggested that it would benefit emergency food providers and recipients for food banks and pantries to “…take a look at their volunteer recruiting process and practices. You know, what other organizations…if they’re finding their volunteers through companies or local businesses because a lot of businesses try to move their employees to volunteer.”

While proposing ways to mitigate food-related health disparities perpetuated by the disconnect between food donors and recipients, some key stakeholders identified narrative storytelling as a potential tool for mobilizing support to alleviate food banking system characteristics that perpetuate diet-related health inequities among clients. One national program officer shared: “We’re looking at how we can elevate the storytelling aspect. We have a pretty robust communications arm, and so, we are taking a look at what we can do and who we can work with to spread and share stories—lift them up—to audiences that may not necessarily know about the issue”.

A State House representative noted that quantitative data—the primary tool used to lobby for policy changes—has its limitations, particularly in the current administration and sociopolitical climate. This interviewee suggested that elevating personal stories and experiences may have the power to humanize the experience of food insecurity and promote meaningful changes in policy and practice.

I heard an advocate last week on a panel about the current political climate and federal policy, and he kind of said that the current administration doesn’t care so much about data. But if you tell them a good story, that may be a little more helpful. And food banks have a million stories because of all of the folks that they interact with. And seeing these—particularly the local food pantries—seeing these people from week to week, understanding what their needs are, what they’re facing, potentially having some really good success stories of how a pantry or a bank can help through other services that the pantry provides—getting folks connected into jobs or getting them on food stamps—may have helped them become less food insecure and moved up the income latter, the economic latter. They have so many stories that probably aren’t being shared as much as they should be to change the perception of those who are food insecure and what that means, not just health-wise but just in general for a family and the reliance on the food bank and pantry system. That would be really useful right now. And certainly, they want to have the data to back up the stories, but I think that this is a good time to start sharing more stories.

#### Access to information

Another theme that emerged across interviews was how very limited access to information about healthy eating and navigating the complexities of the food banking system contributes to obesity and other health disparities among clients. Highlighting the macro-level disparity between food banking system clientele and the broader society regarding knowledge about selecting and preparing healthy foods, one national program officer stated:

The whole local food movement was started among those who were upper and middle-class. Because they could access local, sustainable, organic, all of that. Either through their local grocery store that started selling more organic stuff or through their farmers’ markets. And they have the accessibility, they’re able to use whatever transportation available to them, they have the money and probably also the education. But, also, those who are educated, they may not necessarily be wealthy, but those who are educated about the need to eat more healthfully—and they have the know-how on how to cook those items, those more wholesome items, too. My hairdresser is probably not wealthy, but she knows that she needs to cook, you know, healthfully, and she goes to Trader Joe’s. She has the means and the knowledge to eat better.

Sharing a similar perspective, one food bank director identified poverty as the root cause of these overarching discrepancies in healthy eating awareness:

And, they might not be as well educated on what constitutes a good diet because they haven’t been exposed as much to that information for whatever reason. So, those two to me anyway are the main issues that poverty causes all kinds of problems for people, and certainly, it has an impact on people’s nutritional intake.

A food bank board member noted how even for those who possess the information needed to select and prepare healthy foods, a lack of access to information about navigating the many intricacies of the food banking system may prevent many potential clients from accessing nutritious foods. Language barriers, differences in policy implementation across food banks, and a general understanding of how the food banking system works constitute some of the key barriers preventing those in need from utilizing relevant services.

### The relative impact of impact of improving the food banking system (FBS) vs. other systems

Most key stakeholders expressed that improving characteristics of the FBS would have a substantial impact on alleviating inequitable risks of obesity and hunger among clients. However, a food bank director and a food bank board member expressed skepticism regarding the relative importance of leveraging the FBS for this purpose. These stakeholders expressed uncertainty regarding the relative importance of the FBS to nutrition and health when compared to the impact of other systems, such as local food systems in communities. Their views were rooted in the belief that the FBS does not contribute to a significant proportion of clients’ food intake. For example, the food bank director stated:

I think if you’re looking to the emergency food system to cut down on health disparities in the overall low-income population, I think you’re probably looking in the wrong place because, again, a small percentage of the food they receive is coming [from the food banking system].

The sentiment that food from the FBS does not make up a significant portion of clients’ food intake was intertwined with the view that there are no structural inequities in the FBS. Instead, the food bank director argued, “The fact that there are so many people in poverty is the bigger reason for health inequities.” Key stakeholders also noted that poverty and “-isms,” such as racism and sexism, contribute more to the hunger-obesity paradox, compared to the impact of the FBS. Highlighting poverty as the root cause of hunger, one food bank board member stated:

A lot of this I think is driven by the economics of the environment and whether that’s through minimum wage, whether that’s through creating better educational opportunities so that individuals that are stuck in poverty have the ability to develop the skills so they can move into jobs that have better pay and a better position to not be food insecure. If we could solve some of the economic issues, I think it would help to take care of some of the food insecurity issues that we’re dealing with.

A community-engaged researcher referenced other “-isms” as the root causes of hunger:

Very often when we think about the issue of hunger, we think ‘oh my gosh there isn’t enough food. We need to throw food at the problem.’ Part of the reason people don’t have food is that they don’t have a living wage job, they don’t have affordable healthcare and childcare, etc. To me, these are the root causes. And then if we dig even deeper of why certain people are more at risk, these root causes, then we get into the racism, the sexism, the other–isms. …we really can’t ‘food bank’ our way out of this problem.

## Discussion

The results from this qualitative study of key stakeholders offer useful insights regarding both structural and social characteristics of the U.S. food banking system that potentially contribute to obesity and hunger among its clients. Our research builds upon previous studies with a systems-level focus on food banking and food insecurity, nutrition, or health [30–33] by contributing an intentional focus on a health equity lens that enables identification of overt and subtle injustices that create unfair burdens in a population [42–45, 48–51]. Key findings from this study align with Kumanyika’s equity-oriented obesity prevention framework, which stresses that health disparities-including obesity-cannot be alleviated without addressing underlying inequities like access to social and economic resources [8, 49, 51].

Most key stakeholders believed that efforts to improve the characteristics of the food banking system in the United States were a promising way to alleviate inequitable risks of obesity and hunger among clients over time. However, two key stakeholders expressed uncertainty regarding the importance of leveraging the food banking system for this purpose relative to addressing other societal issues like poverty, racism, or sexism. Both stakeholders justified this position with their view that clients rely less on pantries and more on other types of food outlets to access most of their food. Notably, that perspective is not supported by current evidence [34, 36, 37, 39, 40, 46, 47, 52]. Clients acquire food from pantries as often as they do from grocery stores and more often than they do from dollar stores, supercenters, and convenience stores [36].

Multiple themes emerged from our research drawing linkages between *structural characteristics* of the food banking system and obesity disparities. These include: (a) inadequate access to nutrient-dense food from donors; (b) federal emergency food policy and programming; (c) state-level emergency food policy and programming; (d) geographic variation in access to health-promoting resources; and (e) gaps in food supply versus client need. These findings align with a 2017 National Academies report on community-based solutions to health in the United States [45] that makes a distinction between structural vs. social inequities describing the former as “deeply embedded in the fabric of society”. The authors draw from a variety of settings including the U.S. education, banking, housing, and labor systems to demonstrate the substantial and long-lasting impacts that structural factors (e.g., policy, governance, race, gender) have on health outcomes.

The key stakeholders in our study indicated that disparities in the allocation of obesity prevention resources at the donor level may contribute to some clients being more susceptible to obesity than others. This is consistent with existing literature that documents the FBS’ reliance on inappropriate donations, such as soda, candy, and other unhealthy food products, as a structural factor that influences the overall nutritional quality of foods distributed through the system [30, 53–56]. Our findings highlight the overall gap between the types of foods clients need to support their health and the types of foods available. This aligns with previous studies documenting the importance of providing appropriate options in food pantries for clients with diabetes [57–60]. Also, our findings contribute new information regarding how a nutritionally inadequate food supply underserves and may even exacerbate pre-existing health conditions for clients that are managing obesity and obesity-related conditions (e.g., heart disease).

The relevance of TEFAP to improving the nutritional quality of foods distributed through the food banking system and ultimately clients’ health outcomes is acknowledged but underexplored in the context of promoting equity. Key stakeholders shared how the current sociopolitical climate in the U.S. and local differences in how federal TEFAP ID requirements are implemented and enforced likely contribute to disparate risk for dual experiences of hunger and obesity disparities among foreign-born populations. Further, key stakeholders suggested that placed-based obesity inequities among food pantry clients could be partially linked to some states lacking emergency food programs and to urban vs. rural differences in access to local farms with the capacity to distribute fresh produce to pantries [33].

In addition to structural issues, social and interpersonal challenges are also important. To date, health-related research on attitudes and social dynamics between system leaders and clients has mainly focused on leaders at the pantry-level (e.g., staff, volunteers) [61, 62], clients’ coping strategies [60, 63] and developing client-choice pantries or nutrition interventions [36, 61, 62, 64, 65]. Fewer studies have considered attitudes and behaviors among upstream actors, such as donors and food bank directors, as the current study has done [30, 53, 56, 64, 66]. Our results point to several *social and interpersonal* challenges between system leaders and clients that potentially create and maintain disparate obesity risks in the target population including: (a) stereotypes; (b) language barriers; (c) mistrust in communities of color; (d) lack of diversity/representation among system leaders; and (e) access to information as a privilege.

In the current study, key stakeholders indicated that these social/interpersonal challenges stem from power differentials rooted in the larger context of racial/ethnic power relations in the U.S. Specifically, they pointed out that food banking system leaders are often from the racial/ethnic majority, while their food pantry clients primarily represent racial/ethnic minorities. Key stakeholders further noted the stark differences in the experiences between food insecure versus food secure individuals that result in misunderstanding and mistrust. Misunderstanding and mistrust negatively impact the interactions between system leaders and clients, as they may lead to clients’ hesitation to access the food pantry or associated programs. An example of this misunderstanding is the finding that food pantry staff and leadership assume that clients are not interested in healthier food items [36, 62, 66, 67] leading the food bank or pantry not to prioritize procuring and stocking healthier foods.

Key stakeholders also mentioned the role of the media in exacerbating stereotypes of food insecure individuals by portraying their food insecurity as a sign of their “laziness” or lack of diligence in achieving financial security. They perceived clients’ lack of access to information as another barrier that exacerbates the hunger-obesity paradox among pantry clients. Notably, key stakeholders conceptualized information access as a privilege often reserved for subgroups of clients with some degree of social advantage or power. As a result of their lack of access to information, clients may not be aware of their rights in food pantry settings or take full advantage of the health-promoting resources that they are eligible for in the community, further perpetuating obesity disparities.

### Implications for policy and practice

Kumanyika’s 2019 getting to equity (GTE) framework builds upon Swinburn’s analysis grid for environments linked to obesity which points to potential policy, systems, and environmental (PSE) strategies for improving obesity-promoting environments at the macro- and micro-level by adding specific equity-focused ways of thinking and acting [49]. The GTE framework includes four categories of PSE interventions including: [a] Increasing Healthy Options, [b] Reducing Deterrents, [c] Improving Social and Economic Resources, and [d] Build on Community Capacity. It also includes the scales of justice in the center to reflect the importance of considering synergies across categories. Next, we will draw from this framework to outline several equity-oriented PSE strategies to address the co-occurrence of obesity and hunger of clients of the food banking system in the United States.

#### Increasing healthy options & reducing deterrents

According to the GTE framework, dual efforts to increase access and reduce deterrents to healthy options are critical components of equity-oriented obesity prevention strategies [49]. Inspired by a growing recognition of the substantial impact that the food banking system can have on obesity and obesity-related illnesses, many new policies have been introduced (i.e., Foodbank of Central New York’s “no soda, no candy” policy) and there have been several organizational changes (e.g., inventory tracking systems) [53, 54, 61, 64, 65, 68–71]. Key findings from the present study link unhealthy food donations in the food banking system to imbalanced power between food banks and donors.

Written nutrition policies offer a viable strategy for food banks to increase their power by allowing them to standardize how appropriate and priority food donations are communicated to the donor community. Further, written policies restricting unhealthy food donations may neutralize perceived competition within the food banking system where some food banks may hesitate to strain relationships with donors out of fear that they will lose resources to other food banks. Future efforts should advance equitable access to these viable tools used to increase the distribution of healthy food products while disincentivizing the distribution of unhealthy foods by investing in building support and capacity across the national network of food banks.

Also, in line with the GTE framework, our findings have implications for reducing discrimination and social exclusion as deterrents of health equity among clients. Specifically, organizations within the food banking system may consider examining their hiring processes to ensure that they are proactively recruiting and hiring individuals who are demographically representative of their clients as a way to address the gaps between clients, staff, and other leaders. Such changes have the potential to contribute to clients being able to regularly access food pantries and other distribution sites with more inclusive environments. On a related note, stricter enforcement of Civil rights and Anti-discrimination training for food banking system leaders like individuals that volunteer in TEFAP-accepting pantries may also be warranted [72]. The ultimate goal would be to encourage executives, staff, member agency directors, and volunteers to assess and dismantle unconscious stereotypes and misperceptions of clients.

#### Improving social and economic resources

Disparate access to social and economic resources at both the individual-level (e.g., direct services and referrals) and community-level (e.g., outreach, advocacy) [49] may be another key driver of obesity disparities among populations experiencing food insecurity. During some of the interviews, key stakeholders identified existing programs designed to provide clients the education and support needed to access, select, and prepare healthier foods (e.g., TEFAP, state-level equivalents of TEFAP, SNAP incentives, SNAP-Ed). These findings could be used to bolster existing programs, policies, and practices and enhance their focus on alleviating obesity inequities. For example, emergency food programs’ outreach efforts might be designed to target preexisting resource and information gaps and build trust among clients of the food banking system over time, particularly those from historically marginalized communities. Further, marketing and advertising materials could be increasingly tailored to avoid perpetuating stereotypes of program beneficiaries and to be more accessible and culturally-appropriate to a variety of non-English speaking populations. Last, investing in the expansion of state-level equivalents of the federal TEFAP programs could have a significant impact on advancing equitable allocation of fruit, vegetables, and other health-promoting resources.

#### Build on community capacity

Drawing from the GTE framework, future efforts to improve equity in the food banking system should build upon community capacity among clients [49]. This approach draws from existing research that has investigated clients’ food preferences and found that clients desire more nutrient-dense food options at pantries, including fruits and vegetables [36, 60, 62, 63, 67]. Ultimately, clients should have a voice in decision-making as their health and well-being are directly impacted by the upstream characteristics of the food banking system. This includes the expansion of research and advocacy partnerships like Drexel University’s Witness to Hunger Program which started in 2008 [73, 74]. In particular, participatory action research and citizen science approaches should be promoted as viable strategies for enhancing client representation, inclusion, empowerment, and self-sufficiency in the context of designing, implementing, and evaluating PSE interventions addressing inequitable risks in the co-occurrence of food insecurity and obesity [73–80]. As reflected in key findings from the current study, narrative storytelling is a viable strategy for improving client representation [73]. This includes the donor community which encompasses corporations and individuals that may have limited interactions with people experiencing low food security.

Food banking system leaders will also need to expand and enhance strategic partnerships, particularly in communities that have been underserved historically. For example, Feeding America and Morgan Stanley collaborated on the Healthy Cities Project from August 2014 to May 2015 [64]. This initiative focused on creating strategic partnerships between three food bank sites (i.e., Alameda County Community Food Bank in Oakland, CA; the Greater Chicago Food Depository in Chicago, IL; the Community Food Bank of New Jersey in Newark) and neighboring school-based community programs in low-income areas that offer health-promoting resources like nutrient-dense foods. This successful pilot linking food banks and schools led to the program’s being expanded to Cleveland, Houston, and two additional sites from 2015 to 2018.

In addition to partnering with the education system, the food banking literature provides examples of strategic partnerships with the healthcare system [81–84]; civic organizations focused on social determinants of health like housing and gender equity [77, 78, 80, 81]; and food policy councils as viable options for addressing food insecurity [61]. Regarding the former, a recent systematic review points to a critical need for high-quality evaluations of hospital-based food insecurity intervention, including those with a focus on the co-occurrence of hunger and obesity [84].

### Strengths and limitations

This novel study has several strengths, including a systems focus and data gathered from a diverse set of key stakeholders regarding obesity and food insecurity within the food banking system. The key stakeholders who comprised our sample represent the views of various sectors of the food banking systems including food bank directors, board members, advocates, elected officials, and funders. They were also from a range of geographic locations across the United States. Despite the diversity in the points of view highlighted in this study, several common themes emerged. Both this diversity and the common threads across interviews constitute relative strengths.

Several study limitations are noted. Study participation was limited to a purposive sample of ten key stakeholders in the food banking system. Although the purposive sampling method was well-suited for our study, it may limit the generalizability of these results. Still, recruitment continued until saturation was achieved. Another limitation was that stakeholders may have responded to questions in ways that they deemed socially desirable. However, since the key stakeholders were instructed to respond to questions drawing from their professional experiences in the food banking system, and they voiced many challenges, we do not believe that social desirability was a significant barrier to data quality.

## Conclusion

This qualitative study explored key stakeholders’ perspectives of the relationship between the U.S. food banking system and disparities in the dual burden of hunger and obesity among clients. The study can inform the design, implementation and longitudinal evaluation of large-scale PSE strategies aiming to address health disparities among clients of the food banking system. Future work should utilize mixed-methods and novel participatory action research approaches to better align food bank directors’ and donors’ views with clients’ experiences (beyond coping with food insecurity) and their ideas for how to modify the food banking system to alleviate disparities. These findings will contribute practical solutions for bridging the gap between clients, food banking system leaders, and policymakers with the ultimate goal of achieving health equity among clients of the food banking system.

## Supporting information

S1 Material (NVP)
